# Preliminary Study on Artificial versus Animal-Based Feeding Systems for *Amblyomma* Ticks (Acari: Ixodidae)

**DOI:** 10.3390/microorganisms11051107

**Published:** 2023-04-24

**Authors:** Patrick Stephan Sebastian, Nina Król, María Belén Novoa, Ard Menzo Nijhof, Martin Pfeffer, Santiago Nava, Anna Obiegala

**Affiliations:** 1 Instituto de Investigación de la Cadena Láctea (IdICaL) CONICET-INTA, Rafaela 2300, Argentina; 2 Institute of Animal Hygiene and Veterinary Public Health, University of Leipzig, 04103 Leipzig, Germany; 3 Institute of Parasitology and Tropical Veterinary Medicine, Freie Universität Berlin, 10117 Berlin, Germany

**Keywords:** hard ticks, feeding systems, membrane-based feeding, laboratory animals, 3R principle, South America, Argentina

## Abstract

Hard ticks pose a threat to animal and human health. Active life stages need to feed on a vertebrate host in order to complete their life cycle. To study processes such as tick-pathogen interactions or drug efficacy and pharmacokinetics, it is necessary to maintain tick colonies under defined laboratory conditions, typically using laboratory animals. The aim of this study was to test a membrane-based artificial feeding system (AFS) applicable for *Amblyomma* ticks using *Amblyomma tonelliae* as a biological model. Adult ticks from a laboratory colony were fed in a membrane-based AFS. For comparison, other *A. tonelliae* adults were fed on calf and rabbit. The proportions of attached (AFS: 76%; calf/rabbit: 100%) and engorged females (AFS: 47.4%; calf/rabbit: 100%) in the AFS were significantly lower compared to animal-based feeding (*p* = 0.0265). The engorgement weight of in vitro fed ticks ($\bar{x}$ = 658 mg; SD ± 259.80) did not significantly differ from that of ticks fed on animals (*p* = 0.3272, respectively 0.0947). The proportion of females that oviposited was 100% for all three feeding methods. However, the incubation period of eggs ($\bar{x}$ = 54 days; SD ± 7) was longer in the AFS compared to conventional animal-based feeding (*p* = 0.0014); $\bar{x}$ = 45 days; SD ± 2 in the rabbit and (*p* = 0.0144). $\bar{x}$ = 48 days; SD ± 2 in the calf). Egg cluster hatching ($\bar{x}$ = 41%; SD ± 44.82) was lower in the AFS than in the other feeding methods (rabbit: $\bar{x}$ = 74%; SD ± 20; *p* = 0.0529; calf: $\bar{x}$ = 81%; SD ± 22; *p* = 0.0256). Although the attachment, development, and the hatching of AFS ticks were below those from animal-based feeding, the method may be useful in future experiments. Nevertheless, further experiments with a higher number of tick specimens (including immature life stages) and different attractant stimuli are required to confirm the preliminary results of this study and to evaluate the applicability of AFS for *Amblyomma* ticks as an alternative to animal-based feeding methods.

## 1. Introduction

Hard ticks (Acari: Ixodida: Ixodidae) are one of the most important vectors for arthropod-borne pathogens worldwide. They are able to transmit a wide spectrum of viruses, bacteria, and protozoa to humans and animals. All hard ticks are obligate hematophagous parasites with three active life stages (larva, nymph, and adult—female or male). In the parasitic phase, ticks need to feed on vertebrate hosts once per life stage for at least a few days, and completing their blood meal is crucial for their subsequent development or for oviposition [1].

For studies on ticks and tick-borne pathogen transmission, it is necessary to breed and maintain viable tick colonies under defined laboratory conditions. Hard ticks from laboratory colonies are usually fed on experimental animals such as rabbits, sheep, cattle, and guinea pigs [2]. This requires labor, time, and costly animal maintenance support and needs to be in compliance with ethical standards. In order to contribute to the 3R principle (Reduce, Replace, and Refine) in animal research, the implementation of artificial tick feeding methods offers an interesting alternative to conventional animal-based feeding systems.

The artificial tick feeding methods most widely used can be divided into three groups: (I) capillary feeding, (II) feeding in a semi-artificial system with membranes made of animal skin, and (III) artificial feeding with membranes made of silicone that are used as “skin”, which is the method that requires the least animal usage [3].

Although artificial feeding systems (AFS) are increasingly being used, in vitro feeding systems are far more commonly employed for other hematophagous arthropods, such as mosquitoes, flies, and fleas [4,5,6,7,8,9,10,11,12]. Feeding systems for arthropods other than hard ticks are in general easier to implement as these insects feed on blood for only several seconds or minutes. The establishment of AFS for hard ticks is far more challenging as the feeding period lasts at least a few days and, particularly for adult females, requires larger amounts of blood. Furthermore, other factors such as the complex pre-feeding behavior of ticks [13,14]—which can be complicated to mimic in vitro—make it difficult to establish successful in vitro feeding systems for hard ticks. Moreover, measuring the success of in vitro feeding systems can be a lengthy process due to the long life cycles of most hard tick species [15,16,17].

Most tick AFS were thus far optimized for *Ixodes* spp. [3,8,10]. Only a few studies exploring attempts of artificial feeding for ticks of the genus *Amblyomma* are available [18,19,20,21,22,23,24]. Previously, partial artificial feeding attempts for ticks of the *A. cajennense* complex were published, however ticks were not fed until complete engorgement and further development was not observed [20,22]. *Amblyomma* is one of the most diverse genera of ticks in the world, with 136 valid species [25]. This genus contains several species with medical and veterinary importance, because they are usually parasites of domestic animals and humans and vectors of pathogenic microorganisms [26,27].

The present study had the following objectives: (I) first attempts of development of an AFS for *Amblyomma* adults with a subsequent development of progeny eggs and larvae, using *Amblyomma tonelliae*¸ a tick species from the *Amblyomma cajennense* complex [28], as biological model, and (II) comparison of the feeding and development success of the established AFS with sister colonies of *A. tonelliae* fed on established conventional feeding systems with laboratory rabbits and calves.

## 2. Materials and Methods

### 2.1. Ticks

*Amblyomma tonelliae* adult ticks (approximately three months of age) used for these experiments originated from a laboratory colony maintained at the Instituto Nacional de Tecnología Agropecuaria, Estación Experimental Agropecuaria Rafaela (INTA EEA Rafaela), Rafaela, Santa Fe, Argentina. This colony initially originated from a single female tick that was collected from cattle in 2017 in El Tunal, Salta Province, Argentina. One week prior to the experiment, females and males were placed together in a 1:1 sex ratio in 15 mL centrifuge tubes (25 females and 25 males each). Ticks were randomly divided into three cohorts that were fed on (I) the artificial feeding system (3 months old), (II) a laboratory rabbit (4 months old), or (III) a calf (3 months old). Unfed ticks were kept at 22 °C and 90% RH with a 13:11 h light–dark regime in an incubator (Typ MK (KL) 600, FLOHR Instruments, Nieuwegein, The Netherlands) until further processing. 

### 2.2. Animal-Based Laboratory Tick Feeding

#### 2.2.1. Laboratory Animals

The laboratory rabbit was obtained from the vivarium of the Faculty of Veterinary Science (Universidad Nacional del Litoral, Esperanza, Santa Fe, Argentina) while the calf (approximately four month of age) originated from the breeding area of the INTA EEA Rafaela. Previous tick exposure of animals can be ruled out.

Tick feeding experiments on the three different feeding systems (described below) were not carried out simultaneously as data from the calf and the rabbit cohorts were recorded earlier in the scope of other experiments (see Section 2.3).

#### 2.2.2. Tick Feeding on Laboratory Animals

The feeding of ticks on laboratory animals was carried out at the INTA EEA Rafaela. A rabbit was held in a plastic box with sawdust in the institutional vivarium while one calf was held in the experimental stable. Both animals had permanent access to aliment and water and were frequently cleaned by trained professionals. The rabbit and calf were held under environmental climatic conditions and the natural light–night regime that prevailed in the laboratory during the experiment. The entire trial was conducted in accordance with ethical standards (as described in Section 2.3).

After one week of acclimatization to the environmental conditions, tick feeding was performed by attaching two feeding units (FUs) on the posterior part of the animals (both—rabbit and calf) following the “pill box method” as described by Heyne et al. [29]. Each FU consisted of a coverable plastic tube (height: 2 cm; diameter: 4 cm) and a circle of fabric (diameter: 5.5 cm) that was used to attach the FU onto the animal skin. Before the attachment of the FUs, hair from the posterior part of the animal was clipped with scissors and shaved using a commercial razorblade. Subsequently, two FUs were attached to the animal. One day after attachment of the FUs to the animal, 5 females and 5 males of *A. tonelliae* were placed in each FU. Afterwards, the FUs were examined visually every 6 to 8 h to observe the attachment and feeding process until detachment of the ticks. Engorged female ticks that detached from the animal’s skin were taken out of the FUs, weighed, and kept individually in plastic containers with punctured lids and netting at 22 °C, 90% RH in complete darkness until oviposition. After the detachment of all ticks, the FUs were removed from the animals.

#### 2.2.3. Ethical Statement

Both animals used in this study were part of other experimental trials executed at the INTA EEA Rafaela. Data obtained for this study were collected within the scope of the maintenance of the *A. tonelliae* tick colony (rabbit) or from ticks used as a negative control in another trial examining the efficacy of chemical products used for tick control (calf). All animal experiments were conducted under the permission of the Comité Institucional para el Cuidado y Uso de Animales de Experimentación (CICUAE—Ministerio de Agroindustria; protocol number 11 and 12).

### 2.3. Feeding Ticks on the Artificial Tick Feeding System

#### 2.3.1. Blood Meal Preparation for Tick Feeding on AFS 

Blood was collected from a calf with heparinized tubes (50 I.E./mL), and supplemented with glucose (2 g/L) (WDT, Garbsen, Germany) and gentamicin (5 µg/mL, Merck KGaA, Darmstadt, Germany). To enhance attachment to the feeding membrane, ATP (51 mg/mL, Carl Roth GmbH, Karlsruhe, Germany) was added to the blood right before usage. Blood was used for up to seven days and stored at 6 °C.

#### 2.3.2. Preparation of Tick Feeding Units for AFS

Tick FUs were prepared as described for the “big feeding units” previously published by Król et al. [30] with slight modifications as follows. Silicone membranes were produced, reinforced with lens cleaning paper (Whatman, Maidstone, UK) [7,31], and left to polymerize for at least 120 h at room temperature. The thickness of the membranes was measured using the Inductive Dial Comparator 2000 (Mahr, Göttingen, Germany). Assembled FUs were left for at least 48 h to harden and checked for leakage as described previously [30]. In total 5 FUs with an ascending membrane thickness (200 µm; 230 µm; 240 µm; 250 µm; 300 µm) were used in the current experiment.

#### 2.3.3. Artificial Tick Feeding

All of the below-mentioned procedures were carried out under a laminar airflow hood (Instrumentalia, Buenos Aires, Argentina). Sheep hair extract [7] was applied in two different concentrations (0.525 mg for two FUs with the thinnest membrane and 0.675 mg for the remaining three FUs) to the inner side of the membrane and left to evaporate for 2 h (including 45 min on a heating block at 45 °C) before ticks were placed in the FUs. Each FU contained 5 females and 5 males. The units were sealed with punctured plastic lids and fine mesh. Blood was prewarmed at 38 °C in a water bath before adding it to sterile 6-well (3.1 mL per well) plates. The well plates were likewise prewarmed on a hot plate (XH-2002; C & A Scientific Co., Sterling, VA, USA). FUs with ticks were fixed with sterile rubber rings (Hansa Armaturen GmbH, Stuttgart, Germany) and immersed in blood-filled wells. Six-well plates were replaced by sterile ones containing fresh blood twice a day (every 10–14 h). Before placing FUs into fresh blood, the outer surfaces of FUs and membranes were rinsed with preheated (38 °C) sterile 0.9% NaCl. In order to determine the attachment rate per FU, the number of visible hypostomes puncturing the membranes was recorded twice a day during the blood change. 

#### 2.3.4. Climatic Conditions and Tick Handling during Feeding on AFS

A pilot experiment was conducted at 26 °C, a 90% RH, and a 12:12 h light–night regime, mimicking the natural conditions of the animal-based feeding experiments. As this led to a rapid fungal contamination and death of the ticks on day 7 upon feeding, the authors chose to continue with different climatic conditions as described below.

Well plates with FUs were placed on hot plates (T = 42 °C; Hot Plate 062, Labotect, Göttingen, Germany) on a subjacent shaker (100 rpm, IKA MTS 2/4 digital, Staufen, Germany) at 22 °C, 90% RH, and a 13:11 h light–night regime. FUs were opened every 24 h, starting on the 3rd day of feeding, in order to remove tick feces and detached ticks. In case of visible fungal contamination of the FUs, the netting was replaced and FUs were treated with 10,000 units/mL of nystatin ready-made solution (Sigma Aldrich) as described previously [30]. The feeding experiment lasted 15 days. Engorged female ticks that detached from the membrane were taken out of the units, weighed, and kept individually in plastic containers with punctured lids and netting at 22 °C, 90% RH in complete darkness until oviposition and/or death.

### 2.4. Data Acquisition and Tick Conservation

The following parameters were recorded for female ticks from the three feeding systems: (I) proportion of attached females (PAF); (II) proportion of engorged females (PEF); (III) feeding duration (TOF, time from attachment to detachment); (IV) weight of the engorged females; (V) proportion of females ovipositing (PFO); (VI) pre-oviposition period (POP, time from female detachment until beginning of oviposition); (VII) incubation period of eggs (IP, time from the laying of the first egg until first egg hatched); and (VIII) larvae hatching rate (PEH, proportion of egg clusters hatching; number of hatched larvae/(number of hatched larvae + number of unhatched eggs)). Adult ticks that showed fungal contamination were taken out of the post-feeding incubation chamber immediately and stored at −20 °C.

### 2.5. Statistical Analyses

PAF and PEF were statistically compared between cohorts using Fisher’s exact test with alpha = 0.05. Engorgement weight of female ticks (in mg), TOF, POP, PEH and IP were compared between the three cohorts using the one-tailed Mann–Whitney U test. The significance threshold was set at *p =* 0.05.

## 3. Results

### 3.1. Tick Feeding on Laboratory Animals

All female ticks attached within first 24 h to the calf (n = 10/10) and to the rabbit (n = 10/10), i.e., the PAF was 100% in both systems. All female ticks that attached to the animals reached complete engorgement (PEF: 100%). The TOF for ticks fed on calf varied from 8 to 16 days ($\bar{x}$ = 11 days), while the TOF for females of the rabbit ranged from 9 to 13 days ($\bar{x}$ = 10 days). The weight of the engorged females varied from 628 to 929 mg ($\bar{x}$ = 749 mg) in ticks collected from the calf and from 310 to 691 mg ($\bar{x}$ = 499 mg) in ticks from the rabbit. Further, all females collected from the animals laid eggs (PFO: 100%). The POP was 5 to 9 days ($\bar{x}$ = 7 days) in both cohorts. The IP was 46 to 53 days ($\bar{x}$ = 48 days) for female ticks detached from the calf and 41 to 49 days ($\bar{x}$ = 45 days) for ticks from the rabbit. The PEH was 74% (40–96%) for females fed on the rabbit and 81% (30–99%) for females fed on the calf (Table 1 and Table S1). A preliminary attempt of an AFS for female *A. tonelliae* ticks failed (see Table S2). 

### 3.2. Tick Feeding on Artificial Membrane in Comparison to the Other Two Cohorts

**Figure 1:**
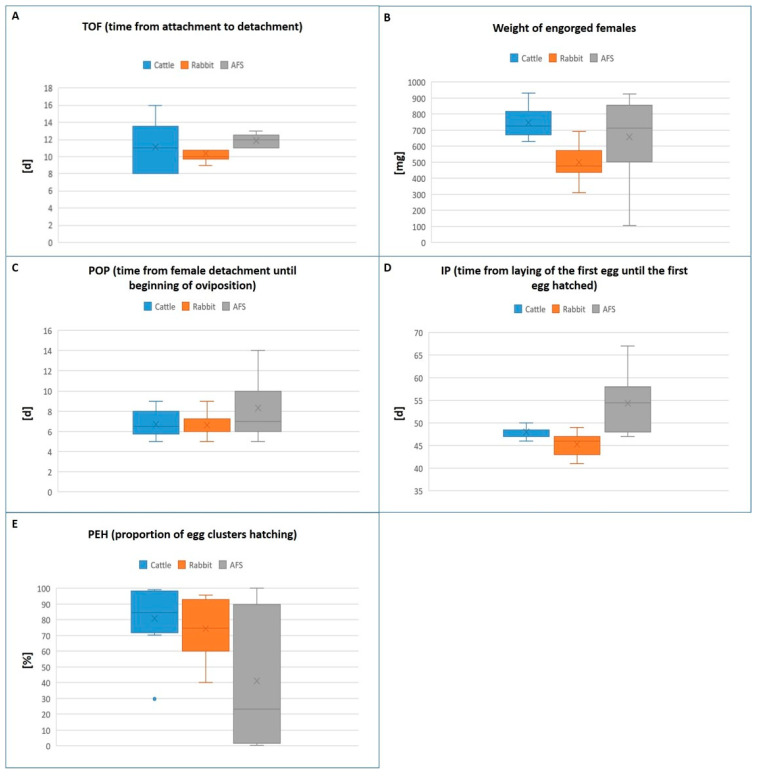
Graphical illustration of the results of the three different feeding systems of female *Amblyomma tonelliae* ticks. (**A**) Feeding duration (TOF, time from attachment to detachment); (**B**) weight of the engorged females; (**C**) pre-oviposition period (POP, time from female detachment until beginning of oviposition); (**D**) incubation period of eggs (IP, time from the laying of the first egg until first egg hatched); (**E**) larvae hatching rate (PEH, proportion of egg clusters hatching; number of hatched larvae/(number of hatched larvae + number of unhatched eggs). [d] = days; [mg] = weight in milligram; [%] = percentage of egg clusters hatching; The line in the middle of each boxplots represents the median; “x” within the boxplot represents the mean value. Dashes represent the minimum and the maximum value of each boxplot; dots represent outliers of each boxplot.

The PAF (76%; 19/25 or 19 out of 25) of the female ticks attached to the membrane within the first 4 days of feeding was significantly lower compared to the cohorts feeding on rabbits and cattle (*p* = 0.0265). The TOF was 13 days on average (12 to 14 days), which was significantly longer than ticks collected from the rabbit but not from the calf (*p* = 0.0131; *p* = 0.4333). The PEF in the artificial feeding system was 47.4% (9/19), which was likewise significantly lower compared to ticks feeding on the calf and rabbit (*p* < 0.0001). The weight of the females that fed and detached in vitro varied from 105 to 924 mg ($\bar{x}$ = 658 mg), which was not significantly different compared to ticks that fed on the rabbit or on the calf (*p* = 0.0947 and *p* = 0.3272). All engorged ticks which survived AFS laid eggs (PFO: 100%). The POP of in vitro fed females was 5 to 14 days ($\bar{x}$ = 7 days), which was not significantly longer compared to ticks feeding on the rabbit or calf (*p* = 0.1009; respectively *p* = 0.1284). The IP was 47 to 67 days ($\bar{x}$ = 54 days) which was significantly longer on average compared to both the rabbit (*p* = 0.0014) and the calf (*p* = 0.0144). All hatched larvae from the three cohorts were vital at least six weeks post hatching. Figure 1A–E show the different developmental values of ticks comparing all three cohorts. The PEH ($\bar{x}$ = 41.18%) was likewise significantly lower compared to ticks collected from the rabbit ($\bar{x}$ = 74.21%) and calf ($\bar{x}$ = 80.84% days) (*p* = 0.0529; *p* = 0.0256).

The PAF ranged between 2 and 5 ticks with a median of 4 ticks per FU in the AFS. The PEF per FU ranged between 2 and 3 with a median of 2 ticks per FU. The difference in membrane thickness per FU resulted neither in different PAF nor PEF.

## 4. Discussion

The use of artificial feeding systems for ticks would be ideal to bypass the use of laboratory animals in laboratory experiments. Previously, partial artificial feeding attempts for ticks of the *A. cajennense* complex were published, however, ticks were not fed until complete engorgement and further development was not observed [20,22]. Here, we describe first successful attempts of using an AFS for the feeding of *A. tonelliae* adults and compare it to the conventional feeding of this species on experimental animals. 

Animal-based feeding usually took 7 to 15 days for *A. tonelliae* females in previous studies [32]. In our study, in vivo fed ticks exhibited similar TOF of 10–11 days on average. Significantly longer TOF were observed for *A. tonelliae* fed in vitro. Reports on TOF of in vitro fed ticks are quite heterogeneous, possibly due to differences in the AFS, blood meal diets, season, tick fitness, as well as differences between tick species and their feeding behavior. 

Ticks usually exhibit prolonged TOF on an AFS when compared to animal-based feeding [3]. In most cases, prolonged TOF may be caused by a longer pre-attachment duration. In this study, the pre-feeding duration was significantly longer (1–4 days) in the AFS compared to conventional animal-based feeding (1 day). Furthermore, frequent disturbance during the blood meal uptake due to the twice daily cleaning of the feeding units can cause intermitting feeding, which could also lead to prolonged feeding durations in AFS [10]. Another reason for these divergent results could be that the surrounding temperature in the in vitro experiment was lower than in the in vivo experiment, which may have led to decreased host-seeking activity in the ticks. Further, previous studies showed that the addition of CO2 to the atmosphere in proximity to the FUs may increase host-seeking activity in AFS [7]. Future experiments should be carried out to evaluate this kind of attractant in connection with tick activity. The PAF and PEF of ticks from the AFS were significantly higher compared to a previous study on *A. cajennense* [20] but lower compared to the in vivo experiments. An insufficient attractiveness of the feeding membrane and the cooler climatic conditions could possibly explain these differences. 

Several attempts have been reported to increase the attractiveness of the artificial membrane, e.g., by adding animal hair extract, tick feces or rubbing the membrane on live animals [18,33,34,35]. For our study, we decided to use sheep hair extract as an attractant only, as other additions might have increased contamination risk [19] which needs to be reduced considering the long TOF of *A. tonelliae* adults in vitro (13 days) in comparison to other hard tick species such as *Ixodes ricinus* (10 days). Most challenging is the possibility of fungal and bacterial growth on the feeding membrane which usually appears after 7 days of in vitro feeding. To further prevent bacterial growth, gentamicin was added to the blood meal. Previous research suggests that the addition of antibiotics to the blood meal may have an influence on the tick microbiome and consequently on tick fitness and fecundity [36,37,38,39]. The use of antibiotics in the blood meal in this study could therefore explain the delayed oviposition and subsequently delayed hatching of larvae compared to the cohorts fed on laboratory animals. Further, it should be borne in mind that the experiments on cattle, rabbits, and on the AFS were not executed simultaneously and that females from the AFS were reared under cooler conditions, which may also have led to delayed oviposition.

The engorgement weight of ticks fed on the AFS was similar to that of the sister colony fed on the calf, which is a natural host for this tick species [26], but slightly higher than the weight of ticks fed on the rabbit. One possible explanation for this observation may be that rabbits are not natural hosts of *A. tonelliae* [26]. However, it was observed for the tick species *I. ricinus* that the blood source does not affect the engorgement weight of the females [40]. The use of natural reservoir hosts as a blood source is the method of choice to obtain conditions that are closest to the physiological reality for tick feeding [41].

The PEH for the ticks fed on artificial membrane ($\bar{x}$ = 41.18%) was likewise significantly lower compared to ticks collected from the rabbit. Habedank and Hiepe [34] report a hatching rate of 49.83% for artificially fed adults of *Dermacentor nuttalli*. The authors suggest that the supplementation of ATP to the blood may result in an increase of the larvae hatching rate. Further studies show that the addition of hemoglobin to the blood meal also may leads to higher hatching rates [42].

Nevertheless, it should be borne in mind that this preliminary study was limited to a low number of tick specimens and only one individual per host species. The experimental design should be adjusted with a higher number of ticks in a more systematic framework and should be repeated with other animals, ideally with other technical staff in order to confirm the results from this study. 

In AFS, it is crucial to adjust the feeding membrane thickness to the tick species it is designed for. Therefore, the length of the tick species hypostome is an important indicator [18]. The hypostome length of *A. tonelliae* females is 0.70 ± 0.05 mm (Nava et al., 2014). In alignment with other AFS the membrane should thus have a thickness of 200–300 µm [18]. In the current study, we compared units with a membrane thickness ranging from 200–300 µm. Membrane thickness did not seem to have an effect on the attachment and the engorgement rates. However, the number of individuals tested was low, requiring testing a higher sample size to make a clear statement and to confirm the first results of this preliminary study. An attempt to feed the hatched larvae on the AFS was not successful).

## 5. Conclusions

This study presents an approach for an artificial feeding system for *Amblyomma* ticks, using *A. tonelliae* as model. Although the PAF, PEF, PFO and PEH were lower than that of ticks fed on laboratory animals, the method may be suitable for studies on tick physiology, pathogen transmission and drug efficacy assays. However, it should be borne in mind that especially in transmission and drug efficacy experiments, there are many factors (for instance pathogen-host interaction or drug metabolism) that may influence the feeding process or success. 

Future studies with a larger number of tick specimens (including immature life stages) and different attractant stimuli are required to confirm the preliminary results of this study. Taking into account that these first results are based on a limited number of tested tick samples, the AFS described here may be a useful alternative for animal-based tick feeding. However, one should keep in mind that results from an AFS cannot be directly extrapolated to ticks in nature.

## Figures and Tables

**Table 1:** Results of three different feeding methods of female *Amblyomma tonelliae* ticks.

Feeding System	Proportion of Attached Females (PAF) [in %]	Proportion of Engorged Females (PEF) [in %]	Time of Feeding (TOF) [in Days] ^1^	Weight of the Engorged Females [in mg] ^1^
Artificial feeding	76 (19/25) ^2^	47.4 (9/19) ^3^	13 (12–14)	658 (105–924)
Rabbit	100 (10/10) ^2^	100 (10/10) ^3^	10 (9–13)	499 (310–691)
Calf	100 (10/10) ^2^	100 (10/10) ^3^	11 (8–16)	747 (628–929)
**Feeding System**	**Proportion of Females Ovipositing (PFO) [in %]**	**Pre-Oviposition Period (POP) [in Days] ^1^**	**Incubation Period of Eggs (IP) [in Days] ^1^**	**Proportion of Egg** **Clusters Hatching (PEH) [in%] ^1^**
Artificial feeding	100 (8/8) ^4,5^	8 (5–14)	54 (47–67)	41 (0–100)
Rabbit	100 (10/10) ^4^	7 (5–9)	45 (41–49)	74 (40–96)
Calf	100 (10/10) ^4^	7 (5–9)	48 (46–53)	81 (30–99)

^1^ mean value (minimum–maximum); ^2^ No. of attached females/No. of total females; ^3^ No. of engorged females/No. of attached females; ^4^ No. of females ovipositing/No. of engorged females; ^5^ One engorged female was excluded from the study due to fungal infection.

## Data Availability

Not applicable.

## Supplementary Materials

The following supporting information can be downloaded at: https://www.mdpi.com/article/10.3390/microorganisms11051107/s1.
