# Supplementary material for: Preliminary Study on Artificial versus Animal-Based Feeding Systems for Amblyomma Ticks (Acari: Ixodidae)
Source: Microorganisms. 2023 Apr 24;11(5):1107. doi: 10.3390/microorganisms11051107 (PMC10221037; doi:10.3390/microorganisms11051107)
Supplement: Supplementary file 1 [file microorganisms-11-01107-s001.zip › microorganisms-2369940-supplementary.pdf]

microorganisms

MDPI

Table S1: Results of the successful feeding of female *Amblyomma tonelliae* ticks by three different feeding systems.

Tick ID	Feeding system	TOF[1] [d]	Weight[2] [mg]	POP[3] [d]	IP[4] [d]	N. of eggs	N. of larvae	Total N. egg cluster	PEH[5] [%]
AFS001	artificial feeding	11	924	7	58	2225	54	2279	2,37
AFS002[6]		12	685	10	-	-	-	-	-
AFS003		12	105	14	51	0	238	238	100,00
AFS004		11	808	7	58	1355	5	1360	0,37
AFS005		12	421	10	48	117	1020	1137	89,71
AFS006		13	781	5	67	2565	38	2603	1,46
AFS007		13	712	5	47	725	480	1205	39,83
AFS008		11	903	7	58	2275	160	2435	6,57
AFS009		12	581	10	48	180	1475	1655	89,12
GC001	calf	8	809,7	9	47	3889	1634	5523	29,59
GC002		8	740,3	8	48	64	6116	6180	98,96
GC003		8	675,3	8	53	1483	4789	6272	76,36
GC004		10	833,1	6	48	1645	4294	5939	72,30
GC005		11	678,7	7	46	111	6979	7090	98,43
GC006		11	659,8	6	47	179	6082	6261	97,14
GC007		11	628,3	6	47	493	5958	6451	92,36
GC008		13	806,8	5	47	1926	4551	6477	70,26
GC009		15	707,5	7	47	1279	3790	5069	74,77
GC010		16	929,2	5	50	130	7085	7215	98,20
CME001	rabbit	9	540,3	7	45	1476	3560	5036	70,69
CME002		9	451,9	8	47	200	4262	4462	95,52
CME003		10	499,6	6	46	538	4760	5298	89,85
CME004		10	548,9	6	43	483	5511	5994	91,94
CME005		10	691,6	6	41	1543	4258	5801	73,40
CME006		10	641,6	6	46	1982	1570	3552	44,20
CME007		10	450,1	9	47	1382	2603	3985	65,32
CME008		10	446,1	7	49	2103	1410	3513	40,14
CME009		13	409,4	5	46	106	2090	2196	95,17
CME010		13	310,4	6	43	896	2814	3710	75,85

[1] TOF = time of feeding; feeding duration (time from attachment to detachment)

[2] weight of the engorged females

[3] POP = pre-oviposition period (time from female detachment until beginning of oviposition)

[4] IP = incubation period of eggs (time from the laying of the first egg until first egg hatched)

[5] PEH = larvae hatching rate (proportion of egg clusters hatching; number of hatched larvae/(number of hatched larvae + number of unhatched eggs)

[6] engorged tick was excluded from the study due to fungal infection

Table S2: Results of a preliminary artificial system feeding female *Amblyomma tonelliae* ticks*.

Feeding system	Proportion of attached females (PAF) [in %]	Proportion of engorged females (PEA) [in %]	Time of feeding (TOF) [in days][1]	Weight of the engorged females [in mg][1,3]
Artificial-feeding	66.67(6/9)[2]	16.67 (1/6)[3]	10 (9-10)	46

* All ticks died during the experiment

[1] mean value (minimum - maximum)

[2] No. of attached females/No. of total females

[3] only one female engorged
